# *Rhynchosia volubilis* Promotes Cell Survival via cAMP-PKA/ERK-CREB Pathway

**DOI:** 10.3390/ph15010073

**Published:** 2022-01-06

**Authors:** Sang-Hyun Ahn, Jung-Soo Suh, Yoon-Kwan Jang, Heon-Su Kim, Gyu-Ho Choi, Eunhye Kim, Tae-Jin Kim

**Affiliations:** 1 Department of Integrated Biological Science, Pusan National University, Pusan 46241, Korea; shahn970114@pusan.ac.kr (S.-H.A.); kem01@pusan.ac.kr (J.-S.S.); ykduke@pusan.ac.kr (Y.-K.J.); heonsu8838@pusan.ac.kr (H.-S.K.); cgh0222@pusan.ac.kr (G.-H.C.); eunhae09@pusan.ac.kr (E.K.); 2 Department of Biological Sciences, Pusan National University, Pusan 46241, Korea; 3 Institute of Systems Biology, Pusan National University, Pusan 46241, Korea

**Keywords:** *Rhynchosia volubilis*, FRET, cAMP, PKA, CREB

## Abstract

*Rhynchosia volubilis*, a small black bean, has been used as a traditional remedy to treat diseases and maintain health in East Asia, but its cellular effects and molecular mechanisms are not fully understood. The purpose of this study was to investigate the effect of ethanol extract from *Rhynchosia volubilis* (EERV) on cell survival and to elucidate the biochemical signaling pathways. Our results showed that EERV stimulated the cyclic AMP (cAMP) signal revealed by a fluorescent protein (FP)-based intensiometric sensor. Using a Förster resonance energy transfer (FRET)-based sensor, we further revealed that EERV could activate PKA and ERK signals, which are downstream effectors of cAMP. In addition, we reported that EERV could induce the phosphorylation of CREB, a key signal for cell survival. Thus, our results suggested that EERV protects against apoptosis by activating the cell survival pathway through the cAMP-PKA/ERK-CREB pathway.

## 1. Introduction

Natural products have long been of interest as treatments for various diseases because of their low cost, high bioavailability, and low cytotoxicity compared to synthetic chemicals [1]. In addition, accumulated evidence suggests that natural products have therapeutic effects on many diseases, such as diabetes, cardiovascular diseases, cancer, and inflammation [2,3,4,5,6]. Among these natural products, soybeans, a healthy nutritional food, have long played an important role as a source of protein and fat in the dietary culture of East Asian countries [7]. They contain various functional ingredients, such as isoflavones, saponins, anthocyanins, tocopherol, and phytic acid [8].

*Rhynchosia volubilis* Loureiro, a small black bean, is a perennial plant that grows in the mountains and fields of Korea, Japan, China, and Vietnam and has been used to treat kidney disease, neuralgia, postmenopausal osteoporosis, and senile dementia [9,10,11,12]. *R. volubilis* extract has exhibited potent antioxidant activity, proliferative effects on human osteoblasts, and anti-obesity efficacy [9,10,13,14,15,16,17]. Further studies revealed that *R. volubilis* contains abundant bioactive constituents, including glycitein, anthocyanin (cyanidin-3-glucoside and delphinidin), flavonoids (quercetin, epicatechin, and apigenin), peptides, and polysaccharides [13,14,18,19,20]. Other studies have also reported that it has nearly 20 times higher isoflavonoids (daidzein, calycosin, biochanin A, and genistein) than other soybeans; thus, it has a functionally positive effect on the human body [21,22]. Most of these compounds are strongly associated with cell survival; however, their molecular mechanisms are not fully understood. 

The goals of this study were to investigate the effect of the ethanol extract of *R. volubilis* (EERV) on cell survival and the underlying molecular mechanism. To this end, we utilized a tool for real-time live-cell imaging using an intensiometric sensor and FRET-based biosensors. Intensiometric sensors have been useful in monitoring the dynamics of intracellular molecules by changing the fluorescence intensity of a single wavelength. Most of these sensors consist of a specific binding domain of a signaling target inserted between two partitions of a fluorescent protein. Conformational switching induced by binding to target molecules results in changes in fluorescence intensity [23,24]. Another type of biosensor used in this study is based on Förster resonance energy transfer (FRET), a nonradiative energy transfer process between donor and acceptor fluorophores [25]. This process depends on the proper spectral overlap of donor emission and acceptor excitation, distance, and relative orientation of the transition dipole moments of the fluorophore [26]. These FRET biosensors have contributed to our understanding of molecular dynamics and signaling pathways with high spatiotemporal resolution at the living single-cell level [27]. Thus, our findings provide important insights for future validation of the efficacy of natural product research and understanding the need for functional studies. 

## 2. Results

### 2.1. Effects of EERV on Cell Viability

**Figure 1:**
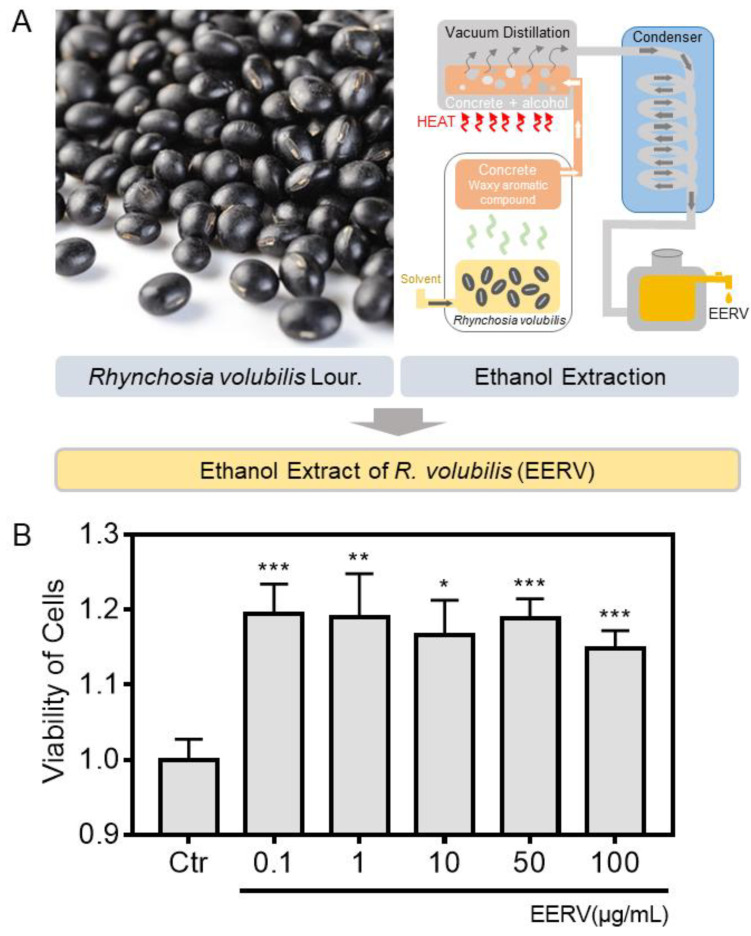
Ethanol extract of *Rhynchosia volubilis* (EERV) and effects of EERV on HeLa cell survival. (**A**) Appearance of *Rhynchosia volubilis* Lour. and production process of ethanol extract of *R. volubilis*. (**B**) Viability of HeLa cells exposed to the control (0.5% (*v*/*v*) DMSO (Biosesang)) and EERV (0.1, 1, 10, 50, and 100 μg/mL) for 24 h, as measured using viability assays. The bar graphs describe mean values of cell viability with error bars indicating the standard error of the mean (S.E.M.) (*n* = 6, * *p* < 0.05, ** *p* < 0.01, and *** *p* < 0.001, Student’s *t*-test). The absorbance values of solubilized formazan product were measured using the Glomax Multi+Microplate Multi Reader (9301-010, Promega, Madison, WI, USA).

A previous study reported that EERV has a cytoprotective effect in a benzalkonium-chloride-induced dye mouse eye model by performing a TUNEL assay, a method for detecting cell death via apoptosis [28]. Moreover, another study showed that EERV affects cell proliferation [15]. Therefore, we performed a WST-8 assay to investigate cell viability according to various concentrations of EERV to verify that EERV has cell survival effects. EERV was used at concentrations of 0.1, 1, 10, 50, and 100 μg/mL for 24 h. The viability of HeLa cells was significantly increased (Figure 1). Although the degree of increase at 100 μg/mL was relatively small, it showed an overall 1.2-fold increase. These data demonstrate that EERV treatment is effective for cell survival (Supplementary Figure S1). 

### 2.2. EERV Induces Intracellular cAMP Concentration Increase 

**Figure 2:**
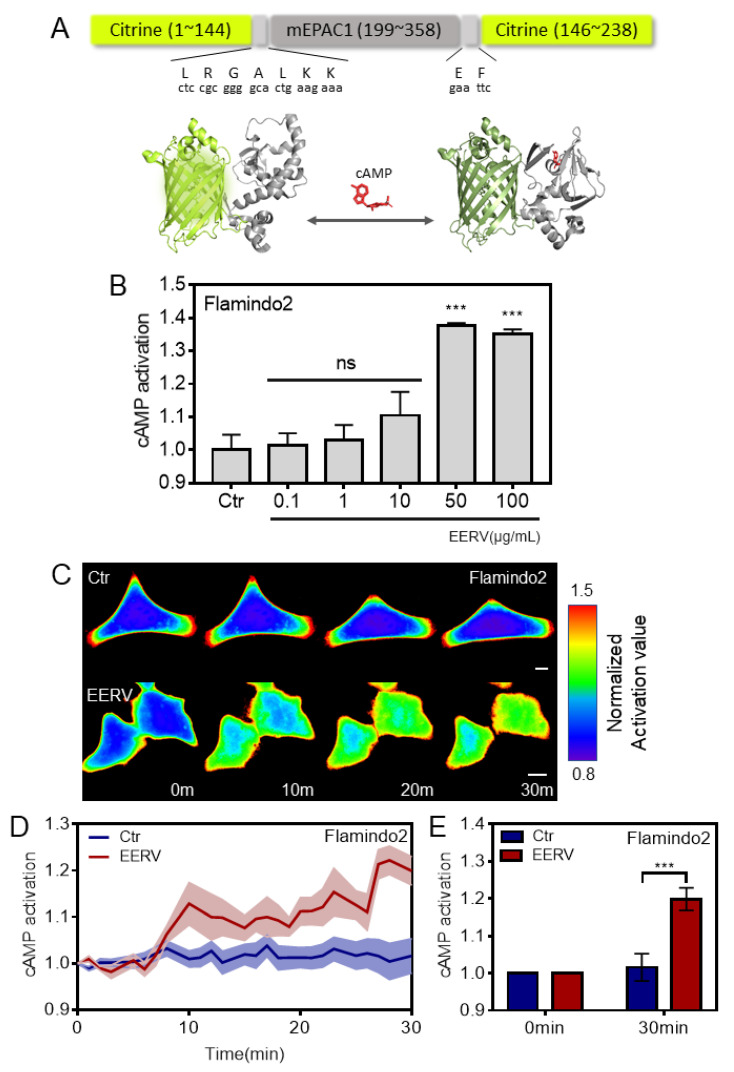
Effect of EERV on cellular cAMP production. (**A**) Domain structure and schematic representation of the intensiometric cAMP sensor, Flamindo2. Three-dimensional representation of unbound (left) and bound (right) Flamindo2 response to cAMP. Images were created using structural graphics for Citrine (PDB 1HUY) and exchange protein activated by cyclic AMP 1 (EPAC1) (cAMP-unbound: PDB 2BYV, cAMP-bound: PDB 4MGK). (**B**–**D**) The effects of EERV on cAMP activity. (**B**) cAMP activation images of the inverse of intensity in HeLa cells exposed to the control (0.5% (*v*/*v*) DMSO), EERV (0.1, 1, 10, 50, and 100 μg/mL) for 1 h. All drugs were treated 5 min after starting live imaging. During the first 5 min, only cells and cell media exist and do not cause changes in the biosensor. Fluorescence values of Flamindo2 detected using the Glomax Multi+Microplate Multi Reader (9301-010, Promega, Madison, WI, USA). The bar graph describes the mean values of normalized activation value, with error bars indicating the S.E.M. (*n* = 9, *** *p* < 0.001, Student’s *t*-test) (**C**) Time-lapse cAMP activation images of the inverse intensity in HeLa cells exposed to the control (0.5%(*v*/*v*) DMSO) and EERV (50 μg/mL). The color scale bars represent the range of the normalized activation value. Hot and cold colors indicate high and low cAMP activity, respectively. Scale bar = 10 μm. (**D**) The time courses represent the average of the change in normalized activation value. The lines are the mean value of the normalized activation value of flamindo2 in HeLa cells treated with 50 μg/mL EERV and DMSO (0.5% (*v*/*v*)) (red and blue; *n* = 9 each). All error bars indicate the S.E.M. (**E**) The bar graph describes the mean values of normalized activation value of flamindo2 at 0 and 30 min. Error bars indicate the S.E.M. (red and blue; *n* = 9, *** *p* < 0.001, Student’s *t*-test).

Previous studies have reported that cellular cAMP concentration is increased by anthocyanins, isoflavonoids, and flavonoids [29,30,31]. Therefore, we hypothesized that EERV might improve cAMP activity because it contains the aforementioned bioactive compounds. To detect the cAMP concentration in the cells, we employed Flamindo2, a cAMP intensiometric biosensor [23]. Flamindo2 is a form of mEPAC1, a binding substrate of cAMP, between the citrine fluorescent protein with 516 nm excitation and 529 nm emission wavelengths. It usually emits light, but when cAMP binds to mEPAC1, it causes a conformational change in the citrine, which results in the loss of light and reduction in the overall intensity of the cells (Figure 2A). The degree of reduction in intensity is expressed as activation by inverse representation for ease of understanding. We performed fluorescence intensity detection to confirm whether cells that overexpressed the flamindo2 biosensor were reduced in intensity because of increased cAMP activation by the EERV treatment (Figure 2B). Treatment with EERV for 1 h at each concentration showed no significant cAMP activation when EERV was used at 0.1, 1, and 10 μg/mL, but showed a significant increase when EERV was used at 50 and 100 μg/mL concentrations. In addition, we performed real-time imaging for 30 min to accurately determine whether the concentration change in cAMP was detected at the single-cell level (Figure 2C). We investigated single-cell real-time imaging by treating EERV at a concentration of 50 g/mL with reference to previous results showing higher cell viability and cAMP activation. At first, imaging was carried out with only cells and media present without any treatment, and DMSO or EERV was treated from 5 min after. The time course graph obtained by this biosensor showed that EERV treatment distinctly induced cAMP activation compared to the control group at the single-cell level. The cAMP concentration increase after 20 min was significant (Figure 2D). These results suggest that EERV plays an essential role in cAMP activation.

### 2.3. EERV-Induced cAMP Regulates PKA and ERK Activation

Our previous results showed that EERV treatment induced a cellular survival effect and increased the cellular cAMP concentration. Therefore, the next question was which effector is activated by cAMP signaling. There are two cAMP downstream effectors, protein kinase A (PKA) and the exchange protein activated by cyclic AMP (EPAC). Many types of downstream effectors have diverse roles [32,33,34]. Therefore, we investigated whether EERV could activate PKA, a potential effector, inducing cell survival according to cellular circumstances. The PKA signaling pathway plays a critical role in regulating a wide range of cellular processes [35,36,37,38]. It regulates the cell cycle and proliferation [39], metabolism [40], transmission of nerve impulses [41], cytoskeleton remodeling [42,43], muscle contraction [44,45], cell survival [46], and other cell processes. One of the most critical targets of PKA is a cAMP-responsive element-binding protein (CREB) [47].

**Figure 3:**
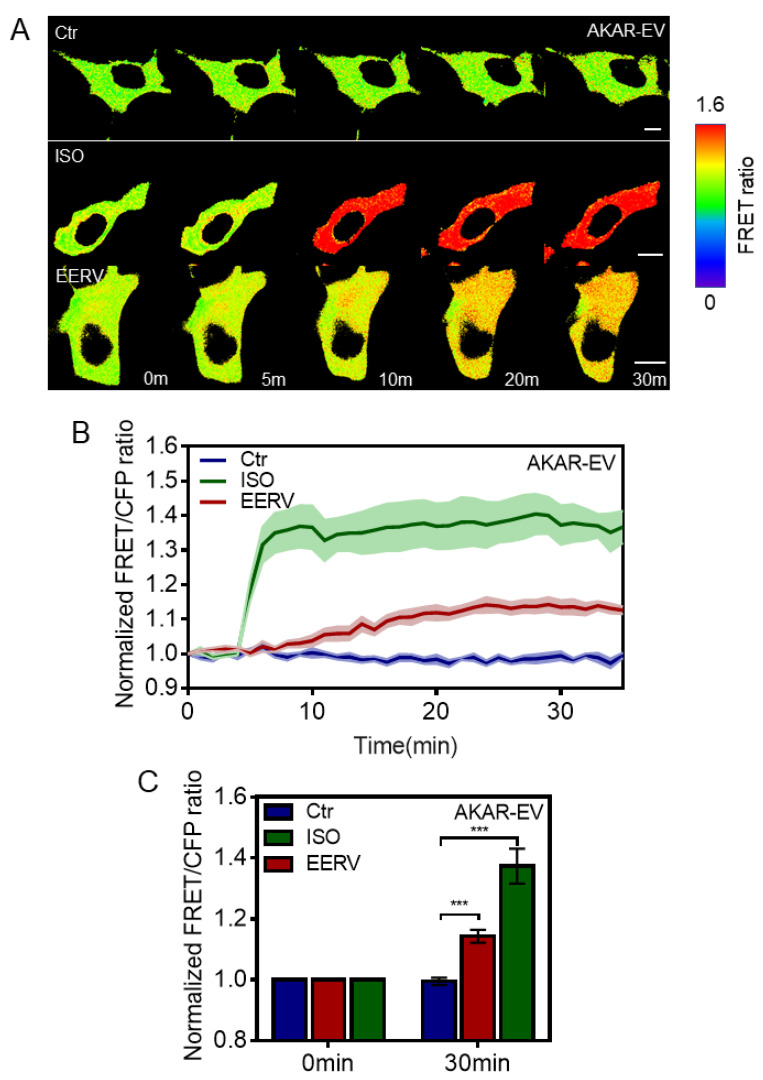
Effects of EERV on PKA activation. (**A**) Time-lapse FRET images of AKAR-EV in HeLa cells exposed to the control (0.5% (*v*/*v*) DMSO), Isoproterenol (ISO, MedChemExpress, 10 μM), and EERV (50 μg/mL). All drugs were treated 5 min after starting live imaging. During the first 5 min, only cells and cell media exist and do not cause changes in the biosensor. ISO was used as a positive control. The color scale bars represent the range of FRET/ECFP emission ratios of the biosensors. Hot and cold colors indicate high and low PKA activity, respectively. Scale bar = 10 μm. (**B**) The time courses represent the average of normalized FRET/ECFP emission ratio changes of EKAR-EV in HeLa cells treated with 10 μM ISO, 50 μg/mL EERV, and DMSO. The lines are the mean values of normalized emission ratios, and error bars indicate the S.E.M. (green, red, and blue; *n* = 5 each). (**C**) The bar graph describes the mean values of normalized FRET/ECFP emission ratios of the biosensor at 0 and 30 min. The bar graph also contains error bars indicating the S.E.M. (green, red, and blue; *n* = 5, *** *p* < 0.001, Student’s *t*-test).

To perform PKA activity real-time imaging, we used the FRET-BRET hybrid PKA biosensor [48]. When the activated PKA phosphorylates the biosensor substrate, the phosphorylated substrate binds to the FHA1 domain, and the distance between ECFP and YPet becomes closer, revealing an increased FRET/ECFP ratio. After transfection with the PKA biosensor and starvation, the cells were treated with DMSO(Biosesang), Isoproterenol (ISO, MedChemExpress, 10 μM), and EERV (50 μg/mL) (Figure 3). At first, imaging was carried out with only cells and media present without any treatment, and each drug was treated from 5 min after. As expected, the time course graph obtained by this biosensor showed that DMSO treatment, used as a negative control, did not exhibit a FRET ratio change. ISO, known as a PKA activator [49,50,51], induces a conformational change in the biosensor and rapidly and dramatically increases the emission signal from YPet. In response to the addition of EERV, the biosensor exhibited a gradual time-dependent increase in the FRET/ECFP ratio (Figure 3A,B). The bar graph shows the normalized FRET/ECFP ratio of the biosensor at 0 and 30 min, indicating that PKA activation by EERV was significant. These results indicated that EERV plays a role in activating PKA and can activate cellular survival-related effectors downstream of PKA.

**Figure 4:**
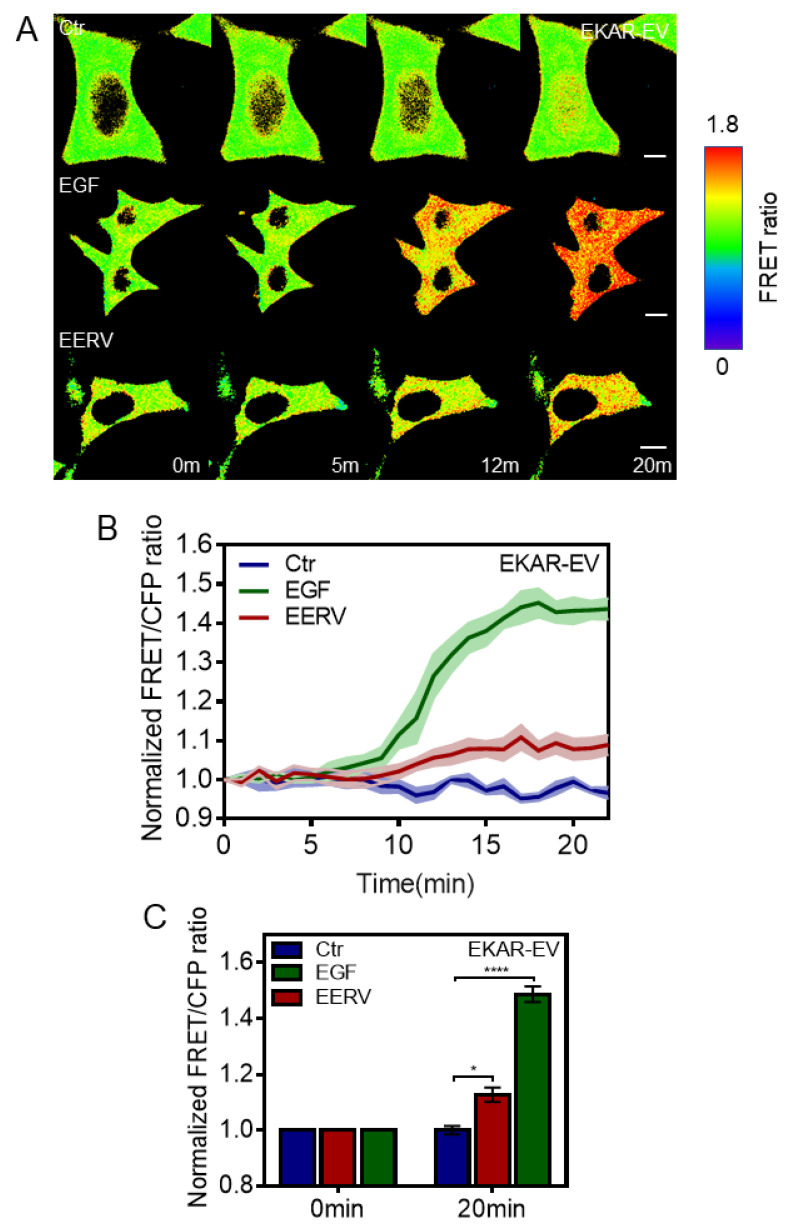
Effects of EERV on ERK activation. (**A**) Time-lapse FRET images of EKAR-EV in HeLa cells exposed to the control (0.5% (*v*/*v*) DMSO), epidermal growth factor (EGF, Sigma-Aldrich, St. Louis, MO, USA, 100 ng/mL), and EERV (50 μg/mL). All drugs were treated 5 min after starting live imaging. During the first 5 min, only cells and cell media exist and do not cause changes in the biosensor. EGF was used as a positive control. The color scale bars represent the range of FRET/ECFP emission ratios of the biosensors. Hot and cold colors indicate high and low ERK activity, respectively. Scale bar = 10 μm. (**B**) The time courses represent the average of normalized FRET/ECFP emission ratio changes of EKAR-EV in HeLa cells treated with 100 ng/mL EGF, 50 μg/mL EERV, and DMSO. The lines are the mean values of normalized emission ratios, and error bars indicate the S.E.M. (green, red, and blue; *n* = 7 each). (**C**) The bar graph describes the mean values of normalized FRET/ECFP emission ratios of the biosensor at 0 and 30 min. The bar graph error bars indicate the S.E.M. (green, red, and blue; *n* = 7, * *p* < 0.05, **** *p* < 0.0001, Student’s *t*-test).

In addition, we investigated the effects of EERV on ERK among various cAMP-Epac downstream effectors (Figure 4). It is generally accepted that cAMP-induced gene transcription is mediated through EPAC and ERK-CREB signaling transduction. It has been reported that anthocyanins and isoflavonoids are abundant in EERV and activate survival effects via the cAMP-ERK-CREB and cAMP-PKA-CREB pathway [52,53,54]. Therefore, we hypothesized that EERV induced ERK activation. The ERK pathway regulates various physiological processes, such as cell growth, proliferation, and survival [55]. It activates ERK by phosphorylation, activation of its kinase activity, and phosphorylation of many downstream targets involved in regulating cell proliferation [56].

When the WW domain detects ERK-specific substrate phosphorylation, the FRET-BRET hybrid ERK biosensor undergoes a conformational change, and the distance between ECFP and YPet is reduced, revealing an increased FRET/ECFP ratio. After transfection with the ERK biosensor and starvation, the cells were exposed to DMSO, epidermal growth factor (EGF, Sigma-Aldrich, 100 ng/mL), and EERV (50 μg/mL) (Figure 4). At first, imaging was carried out with only cells and media present without any treatment, and each drug was treated from 5 min after. As expected, time-lapse images and time course graphs showed that cells in the DMSO, used as a negative control group, did not show any FRET ratio change. Moreover, EGF, used as a positive control, induced the conformational change of the biosensor and rapidly and dramatically increased the emission signal from YPet. In response to EERV addition, the biosensor exhibited a gradual time-dependent increase in the FRET/ECFP ratio (Figure 4A,B). The bar graph, which describes the normalized FRET/ECFP ratio of the biosensor at 0 and 20 min, indicates that ERK activation by EERV is significant. These observations are in agreement with previous studies showing EERV activates the ERK-CREB pathway.

### 2.4. Increasing CREB Phosphorylation by EERV

**Figure 5:**
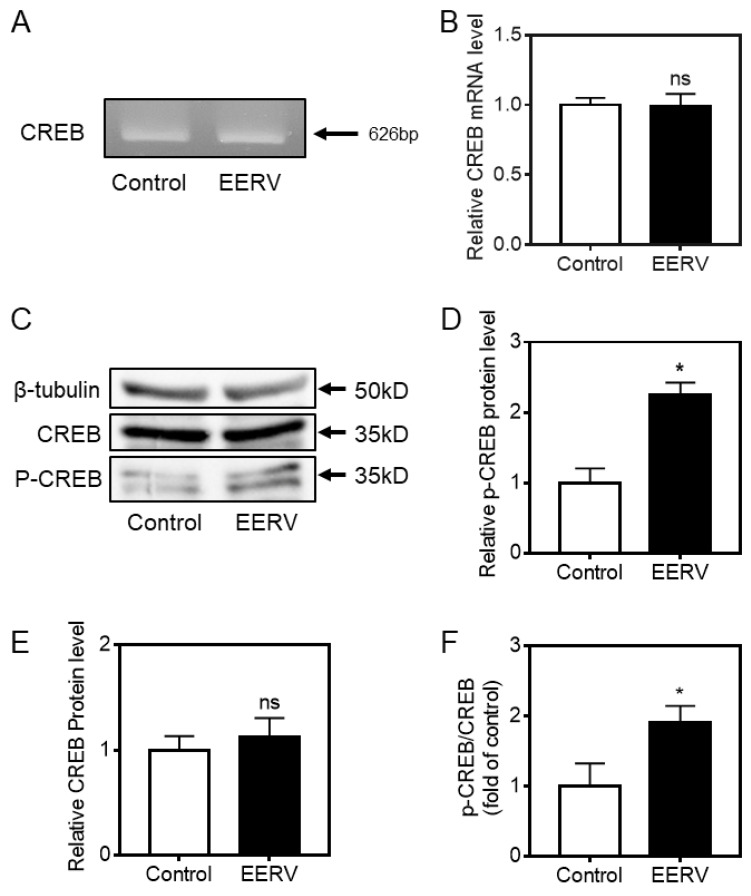
Effects of EERV on phosphorylated CREB activation. (**A**) RT-PCR assay shows mRNA expression levels of CREB in HeLa cells exposed to the control (0.5% (*v*/*v*) DMSO) and EERV (50 μg/mL) for 24 h. (**B**) The bar graphs represent mean values of relative CREB mRNA expression, and error bars indicate the S.E.M. (white and black; *n* = 3 each). (**C**) Phosphorylation of CREB was analyzed by Western blot with phospho-CREB antibodies and relative to total CREB protein after normalization to β-tubulin levels. Protein expression levels of p-CREB, CREB, and β-tubulin in HeLa cells exposed to control (0.5% (*v*/*v*) DMSO) and EERV (50 μg/mL) for 24 h. (**D**–**F**) The bar graph represents the mean values of relative p-CREB, CREB protein expression, and p-CREB/CREB expression ratios with error bars indicating the S.E.M. (white and black; *n* = 3 each, * *p* < 0.05, Student’s *t*-test). Immunoreactive protein bands were detected using a 1:1 mixture of ProNA^TM^ ECL Ottimo A and B (TransLab, Daejeon, Republic of Korea) and measured densitometrically using an iBright^TM^ FL 1500 Imaging System (A44241, Invitrogen, Carlsbad, CA, USA).

We investigated how PKA and ERK activities are responsible for cell survival. Among the downstream candidates, CREB is considered to be a crucial transcriptional factor affected by PKA and ERK [47,57]. This transcription factor is essential to promote neuronal plasticity, memory formation, and neuronal survival [36]. CREB proteins are activated by phosphorylation of various kinases, including PKA, ERK, and Ca^2+^/calmodulin-dependent protein kinases on the serine 133 residue [58]. When activated, the CREB protein recruits other transcriptional coactivators to bind to specific DNA sequences called cAMP response elements (CRE), thereby increasing or decreasing the transcription of the genes [35]. We first scraped cells treated with EERV (50 μg/mL) for 24 h and used them in RT-PCR to verify that EERV increased mRNA expression of CREB itself (Figure 5A,B). After EERV treatment, the mRNA expression level of CREB remained unchanged, suggesting an increase in CREB phosphorylation through the PKA/ERK pathway. Then, we performed Western blotting using cells treated with EERV (50 μg/mL) for 24 h. As expected from the RT-PCR results, the total CREB protein expression level did not change compared to the control group, but the phosphorylated CREB protein level increased (Figure 5C). The bar graph, which describes normalized CREB and p-CREB protein expression levels, shows that p-CREB increases against total CREB expression (Figure 5D–F). These observations agree with previous results showing that the cAMP-PKA/ERK pathway activates p-CREB independent of total CREB expression.

### 2.5. EERV Does Not Activate Adrenoceptor Beta-2

**Figure 6:**
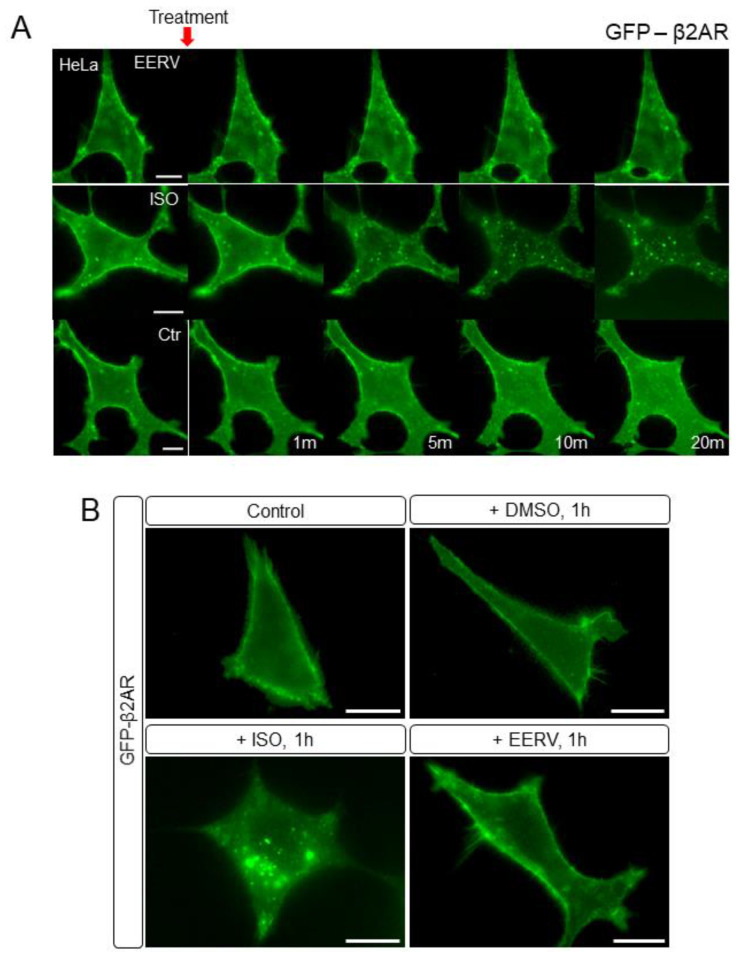
No effect of EERV on adrenoceptor beta-2 activation and internalization in short term. (**A**) Time-lapse fluorescence images of GFP-adrenoceptor beta-2 in HeLa cells exposed to control (0.5% (*v*/*v*) DMSO), Isoproterenol (ISO, 10μM), and ethanol extract of *R. volubilis* (EERV, 50μg/mL). ISO was used as a positive control. (**B**) Fluorescence images of GFP-adrenoceptor beta-2 at 1 h before and after in HeLa cells exposed to control (0.5% (*v*/*v*) DMSO), Isoproterenol (ISO, 10μM), and EERV (50μg/mL). EERV-treated HeLa cells showed no change in the dynamics of adrenoceptor beta-2 compared to ISO group for 20 min and for 1 h.

**Figure 7:**
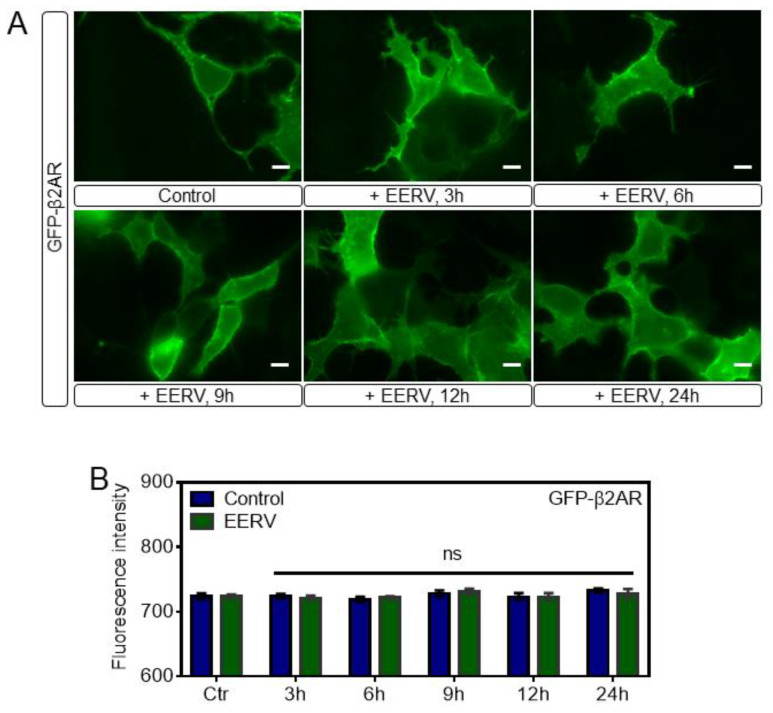
No significant change in the expression level and internalization of the adrenoceptor beta-2 with extended EERV treatment. (**A**) Time-lapse fluorescence images of GFP-adrenoceptor beta-2 at 3, 6, 9, 12, and 24 h in HeLa cells exposed to ethanol extract of *R. volubilis* (EERV, 50 μg/mL). EERV-treated HeLa cells showed no change in the dynamics of adrenoceptor beta-2. (**B**) The bar graph represents the mean values of fluorescence intensity of GFP-adrenoceptor beta-2 at 3 to 24 h. The bar graph also contains error bars indicating the S.E.M. (blue and green; *n* = 8). There were no additional changes in fluorescence intensity of the EERV group for 24 h compared to the control.

We confirmed that EERV increased cell survival effect through cAMP-PKA/ERK-CREB pathway signaling, and we hypothesized that EERV-induced cell survival might be initiated by activating membrane receptors [59,60,61]. This is because many membrane receptors are activated by hormones, and many natural products are similar to the structure of these hormones and thus affect the human body through cell signaling activation through receptor stimulation. Among the many membrane receptors, we investigated whether EERV induces cell survival by binding to the adrenoceptor beta-2 because it is one of the most representative GPCRs and is highly correlated with cell survival [62,63,64]. Adrenoceptor beta-2 is a transmembrane protein that interacts with epinephrine (adrenaline), a hormone and neurotransmitter whose signaling, via adenylate cyclase stimulation through trimeric Gs protein, increased the cAMP production [65]. This activated adrenoceptor beta-2 undergoes internalization into the cytoplasm and internalization has been regarded as a major phenomenon that determines whether the receptor is activated or not [66,67,68]. We investigated whether the expression level of adrenoceptor beta-2 present in cells or internalization occurred when EERV was treated. After transfection with the GFP-adrenoceptor beta-2 and starvation, the cells were exposed to DMSO, ISO (10 μM), and EERV (50 μg/mL) (Figure 6A). At first, imaging was carried out with only cells and media present without any treatment, and each drug was treated from 5 min after. Live imaging was performed for 20 min but, contrary to our expectation, no internalization of the receptor predicting GPCR-ligand binding was observed compared with the positive control, ISO. Adrenoceptor beta-2 changes in cells were observed after 1, 3, 6, 9, 12, and 24 h, but no change was observed (Figure 6B and Figure 7A). In addition, the expression level of GFP-adrenoceptor beta-2 was compared by measuring the fluorescence intensity of GFP in one cell over time, but there was no significant change in the expression level of adrenoceptor beta-2 with EERV treatment compared with the control group (Figure 7B). These results suggest that EERV may affect downstream signaling rather than binding to the receptor.

## 3. Discussion

Natural substances have traditionally been used as health foods and therapeutics in East Asian cultures [69]. These substances have often been shown to improve the survival effects on cells in our body while producing anti-aging, anti-obesity, anti-inflammatory, antioxidant, and neuronal survival effects. For this reason, many studies on natural products and their ingredients are being conducted to determine those beneficial to health with minimal side effects [2,3,4,5,6]. *R. volubilis* is known to have antioxidant, anti-aging, and tissue repair activity [19], and several studies have reported its hormonal action as a substitute for estrogen deficiency [59,60,61]. 

The assay for cell viability and cAMP measurement showed that the survival effect of EERV could be related to cAMP activation. The activation of cAMP and its downstream effectors, PKA and ERK, by EERV treatment was monitored by intensiometric and FRET-based biosensors, respectively. Both methods are versatile and elaborate tools for qualitative and quantitative analysis of protein interactions. In particular, they have the advantage of providing accurate information through real-time spatiotemporal data in living cells, which is highly beneficial for verifying the efficacy and activity of drugs. Access to a wide range of fluorescent materials, in conjunction with improved, easy-to-use, and yet very sophisticated microscopes and spectrometers, has made FRET a prominent technique for biosensing [70]. The unique ability of FRET to probe nanoscale inter- and intramolecular separation distances has also led to a rapidly growing field of structural FRET studies of biomolecules and biological complexes [71]. 

**Figure 8:**
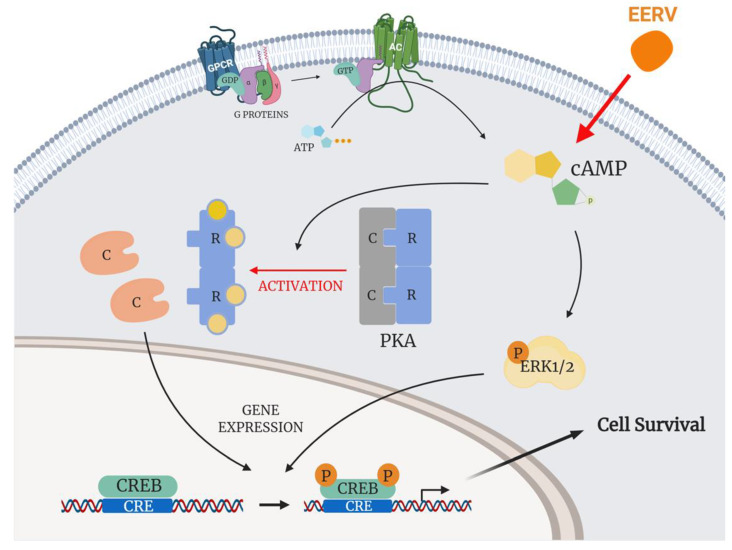
A proposed model of the EERV-induced cell survival pathway. EERV could activate the cAMP-PKA/ERK-CREB signaling axis. This figure was created using BioRender (Available online: http://biorender.com (accessed on 4 February 2021)).

Because the standardization of *R. volubilis* has been reported in previous studies [28,72] and it was revealed that the active compounds of *R. volubilis* were mostly anthocyanins and isoflavonoids, we determined whether EERV could induce cell survival through CREB-associated signaling and its upstream regulators. We found that EERV promoted cellular survival via the cAMP-PKA/ERK pathway (Figure 8). We further confirmed that CREB phosphorylation was involved in EERV-induced cellular survival. Anthocyanins, a component of *R. volubilis*, have extremely strong antioxidant activity [73,74]. Previous studies have reported various bioactivities related to human health, including anti-aging [75], anti-inflammatory [76], and antibacterial properties [77], mitigated visual fatigue, and enhanced blood lipid metabolism [78,79]. Additional studies of the survival effects of anthocyanins have reported that anthocyanin and its dominant component, cyanidin-3-O-glucoside, activate the PKA/ERK-CREB pathway [52,53,54]. CREB is a transcription factor with multiple functions and plays a critical role in cell survival [80,81,82,83]. CREB activation occurs via phosphorylation by various kinases, including PKA and MAPKs [58,84]. In addition, isoflavonoids and flavonoids, the other components of *R. volubilis*, have been previously shown to have survival effects via CREB activation [85,86]. 

## 4. Materials and Methods

### 4.1. Cell Culture and Reagents

HeLa cells from ATCC were cultured in Dulbecco’s modified Eagle’s medium (DMEM, Gibco, Waltham, MA, USA) supplemented with 10% (*vol*/*vol*) fetal bovine serum (FBS, HyClone, Logan, UT, USA), 2 mM L-glutamine, 1 unit/mL penicillin, 100 µg/mL streptomycin (GenDEPOT, Katy, TX, USA), 1 mM sodium pyruvate, and 0.04 mM phenol red. The cells were maintained in a humidified incubator with 95% air and 5% CO_2_ at 37 °C. Cells were transfected at 60–80% confluence with Lipofectamine 3000 (Invitrogen, Carlsbad, CA, USA), according to the manufacturer’s instructions.

### 4.2. DNA Construction and Plasmids

Flamindo2 was previously developed and described by Haruki Odaka (Waseda Bioscience Research Institute in Singapore, Singapore) (#73938, Addgene). Fluorescence intensity analysis was performed as previously described [23]. AKAR-EV and EKAR-EV are FRET-BRET hybrid biosensors that were kindly provided by Naoki Komatsu (Laboratory of Bioimaging and Cell Signaling, Kyoto University, Kyoto, Japan) (AKAR-EV: #108655, https://www.addgene.org/108655/, Addgene, accessed on 27 December 2021), (EKAR-EV: #108652, https://www.addgene.org/108652/, Addgene, accessed on 27 December 2021). These sensors contain two fluorescent proteins that can induce FRET and also contain the bioluminescent protein Renilla luciferase (RLuc8). Therefore, these can be used as FRET biosensors or BRET biosensors depending on the purpose of the experiment. We used both sensors only for FRET imaging in this study. AKAR-EV comprises a FHA1 domain, flexible EV linker, and PKA-specific substrate localized between the FRET donor, ECFP, and acceptor, YPet. EKAR-EV comprises a WW domain, flexible EV linker, and ERK-specific substrate localized between the FRET donor, ECFP, and acceptor, YPet. Each sensor domain binds to a sensor substrate phosphorylated by PKA or ERK. Thus, YPet and ECFP, which are attached to both ends of the sensor, become close, and FRET occurs. That is, in the normal state, 434 nm light is excited and 477 nm light is emitted, but in the active state, 530 nm light is emitted. FRET analysis was performed as previously described [48]. A green fluorescent protein (GFP)-tagged version of Adrenoceptor beta-2 has been well-described in previous reports [87,88].

### 4.3. Plant Material and Chemicals

*R. volubilis* seeds were provided by the Highland Agriculture Research Center, National Institute of Crop Science, Pyeongchang, South Korea. *R. volubilis* was checked at http://www.theplantlist.org (Record 38957) (accessed on 14 October 2019). A voucher specimen (KISTGN-RN-2016-003) was deposited at the KIST Gangneung Institute. Dried *R. volubilis* seeds were ground into powder (1 kg) and extracted twice with 5 L of 70% ethanol at 25 °C for 3 h in an ultrasonic cleaning bath (Model RK 158s, Bandelin, Berlin, Germany) (Figure 1A). The extracts were filtered through Whatman No. 1 filter paper, and the combined filtrate was concentrated to dryness by rotary evaporation at 40 °C to obtain 50.4 g of EERV. Then, the EERV was stored at −20 °C and dissolved in dimethyl sulfoxide (DMSO; Biosesang, Seongnam, Republic of Korea) prior to cell treatment. Epidermal growth factor (EGF) was purchased from Sigma-Aldrich (St. Louis, MO, USA). Isoproterenol (ISO) was obtained from MedChemExpress (Monmouth Junction, NJ, USA).

### 4.4. Viability Assay

Cell viability was determined using WST-8 assays. HeLa cells were seeded at 2 × 10^4^ cells/well in 96-well plates and incubated for 24 h at 37 °C before cells were treated with DMEM containing the control ((0.5%) DMSO) or EERV (0.1–100 µg/mL) with/without several drugs for 24 h. After washing, the cells were exposed to a 9.09% (*v*/*v*) Cellrix^®^ Viability Assay Kit in DMEM without phenol red (31053028) (Gibco, Waltham, MA, USA) for 2 h at 37 °C. The optical density of the solubilized formazan product was measured using a Glomax Multi+Microplate Multi Reader (9301-010; Promega, USA) at 450 nm wavelength.

### 4.5. Fluorescence Intensity Detection

Intracellular cyclic adenosine 3′,5′-monophosphate (cAMP) concentration was detected using Flamindo2, an intensiometric sensor for cAMP. The intensity of YPet decreased when cAMP concentration was high. HeLa cells were seeded at 2 × 10^4^ cells/well in 96-well plates and incubated for 24 h at 37 °C before Flamindo2 transfection. After being starved with DMEM containing 0.5% FBS for 18–24 h, DMEM containing the control (0.5% DMSO) or EERV (0.1–100 µg/mL) was used to treat cells for 24 h. Next, the cells were washed, and the medium was changed with DMEM without phenol red, and the fluorescence was analyzed. Fluorescence of YPet by Flamindo2 was measured using a Glomax Multi+Microplate Multi Reader (9301-010, Promega, Madison, WI, USA) at 490 nm excitation and 510–570 nm emission. The fluorescence intensity measurement of GFP-adrenoceptor beta-2 was also performed in the same method of Flamindo2.

### 4.6. Image Acquisition and Microscopy

Cells expressing several exogenous proteins were cultured in a Confocal Dish (100350, SPL, Yongin, Republic of Korea) and starved with DMEM containing 0.5% FBS for 18–24 h before the imaging experiment. Before the experiment, the cells were washed using phosphate-buffered saline (PBS, LB004-02, WELGENE, Gyeongsan, Republic of Korea), and the medium was replaced with a CO_2_-independent medium (18045-088, Gibco, Waltham, MA, USA) containing 0.5% FBS. Flamindo2 and GFP-adrenoceptor beta-2 images were obtained using a Leica DMi8 microscope equipped with a charge-coupled device (CCD) camera (DFC450C, Leica, Germany), a 436/20 excitation filter, a 455 dichroic mirror, and a 535/30 emission filter. Two FRET images were obtained with two emission filters controlled by a filter changer (480/40 for ECFP and 535/30 for YPet), unlike the intensity images. Las X software (Leica Germany) was used to acquire images and compute the emission intensity of YPet for Flamindo2 images and ECFP and FRET for FRET sensor images. A specific region of the target cells was selected as the region of interest (ROI) to observe signals and conduct the quantification. The fluorescence intensity in the background region was selected and quantified to remove the signal from the ROI of the YPet, ECFP, and FRET channels. Quantified values were analyzed using GraphPad Prism 7.0 (San Diego, CA, USA).

### 4.7. RT-PCR Experiment

After DMSO or EERV treatment, total RNA (100–300 ng) was isolated using TransZol Up Reagent (Transgene Biotech, Haidian District, Beijing, China) according to the manufacturer’s instructions. Subsequently, approximately 300 ng of total RNA from each sample was converted to cDNA using a SmartGene Compact cDNA Synthesis Kit (SmartGene, Lausanne, Switzerland). The resulting cDNA sample served as a template for RT-PCR using the Phusion High-Fidelity PCR Kit (Thermo Fisher Scientific, Rockford, IL, USA), following the manufacturer’s protocol. For RT-PCR amplification of CREB, an initial amplification using CREB primers (forward: 5ʹ-AACACCAACTGGAGAGAGTCA-3ʹ, reverse: 5ʹ-AGTAGACTCTTGCCACGACA-3ʹ) was performed with a denaturation step at 98 °C for 30 s, followed by 35 cycles of denaturation at 98 °C for 10 s, primer annealing at 54 °C for 20 s, and primer extension at 72 °C for 20 s. Upon completion of the cycling steps, a final extension at 72 °C for 7 min was performed, and then the product was stored at 4 °C. The expression levels of target gene transcripts were determined using gel electrophoresis and normalized to the control using ImageJ software.

### 4.8. Western Blot Experiment

After EERV treatment and incubation, cells were washed with cold PBS (LB004-02, WELGENE, Gyeongsan, Republic of Korea), lysed with the CETi Lysis Buffer with Inhibitors (TLP-121CETi, TransLab, Republic of Korea), and centrifuged at 15,000× *g* for 10 min at 4 °C. Total protein concentration was determined using a Pierce^TM^ BCA Protein Assay Kit (23227, Thermo Fisher Scientific, Rockford, IL, USA) following the manufacturer’s protocol. Each sample was added to a 5X SDS-PAGE sample buffer (TLP102.1, TransLab, Republic of Korea) and heated at 100 °C for 5 min. Proteins (20 µg/lane) were loaded onto a 10% SDS-polyacrylamide gel, subjected to electrophoresis, and transferred to an Immobilon-P PVDF Membrane (IPNH00010, Merck Millipore, Burlington, MA, USA). The membranes were blocked in 5%(*w*/*v*) FBS at pH 7.5 pBST with 0.05% Tween 20 (TransLab, Republic of Korea) for 1 h at room temperature. Membranes were incubated with the following primary antibodies in TBS (TransLab, Republic of Korea), each diluted 1: 1000 anti-phospho-CREB (44-297G, Thermo Fisher Scientific, Rockford, IL, USA), anti-CREB (sc-377154, Santa Cruz, Oregon, CA, USA), and anti-GAPDH (HC301, Transgene, Beijing, China) overnight at 4 °C. The membranes were washed three times with PBST and incubated with the appropriate horseradish peroxidase-conjugated IgG secondary antibodies (Santa Cruz Biotechnology, Oregon, CA, USA), diluted 1: 2000 at room temperature for 90 min. Immunoreactive protein bands were detected using a 1:1 mixture of ProNA^TM^ ECL Ottimo A and B (TransLab, Republic of Korea) and measured densitometrically using an iBright^TM^ FL 1500 Imaging System (A44241, Invitrogen, Carlsbad, CA, USA) and ImageJ software. GAPDG expression was detected as a loading control.

### 4.9. Statistical Analysis

Representative images and graphs were obtained from at least three independent experiments. All results are expressed as mean ± standard error of the mean (SEM). Statistical analyses were performed using the unpaired Student’s *t*-test to determine the statistical significance of the difference between the two mean values. Statistical significance was set at *p* < 0.05.

## 5. Conclusions

In conclusion, our results suggest that EERV has a protective effect against apoptosis by activating the cell survival pathway through cAMP-PKA/ERK-CREB. Therefore, our study provides a new experimental methodology for studying the biochemical signaling processes of natural products and provides essential information for understanding physiological activity and cellular function.

## Data Availability

The data presented in this study are available in this article.

## Supplementary Materials

The following supporting information can be downloaded at: https://www.mdpi.com/article/10.3390/ph15010073/s1, Figure S1: Dose-response curve of HeLa cell viability according to the concentration of ethanol extract of *Rhynchosia Volubilis* (EERV). Viability of HeLa cells exposed to the control (0.5% (*v*/*v*) DMSO(Biosesang)) and EERV (0.1, 1, 10, and 100 μg/mL) for 24 h, as measured using viability assays. The data in Figure 1B was used; the cell viability data of each point was replaced with the response value (% max) and is shown by normalizing the maximum value to 100% and the control value to 0% (EC50 = 0.05) (*n* = 6). The absorbance values of solubilized formazan product were measured using the Glomax Multi+Microplate Multi Reader (9301-010, Promega, USA). The graph was generated with GraphPad Prism 7.0 (San Diego, CA, USA).
