# Supplementary material for: Rhynchosia volubilis Promotes Cell Survival via cAMP-PKA/ERK-CREB Pathway
Source: Pharmaceuticals (Basel). 2022 Jan 6;15(1):73. doi: 10.3390/ph15010073 (PMC8778899; doi:10.3390/ph15010073)
Supplement: Supplementary file 1 [file pharmaceuticals-15-00073-s001.zip › pharmaceuticals-1492051-supplementary.pdf]

Supplementary Material

Rhynchosia volubilis promotes cell survival via cAMP-PKA/ERK-CREB pathway

Sang-Hyun Ahn[1], Jung-Soo Suh[1], Yoon-Kwan Jang[1], Heon-Su Kim[1], Gyu-ho Choi[1], Eunhye Kim[1] and Tae-Jin Kim[1,2,3]*

[1]Department of Integrated Biological Science

[2]Department of Biological Sciences

[3]Institute of Systems Biology, Pusan National University, Pusan 46241, Republic of Korea,

*To whom correspondence should be addressed:

Tae-Jin Kim, Ph.D.

Email: tjkim77@pusan.ac.kr

Tel: 82-051-510-2261

Figure S1

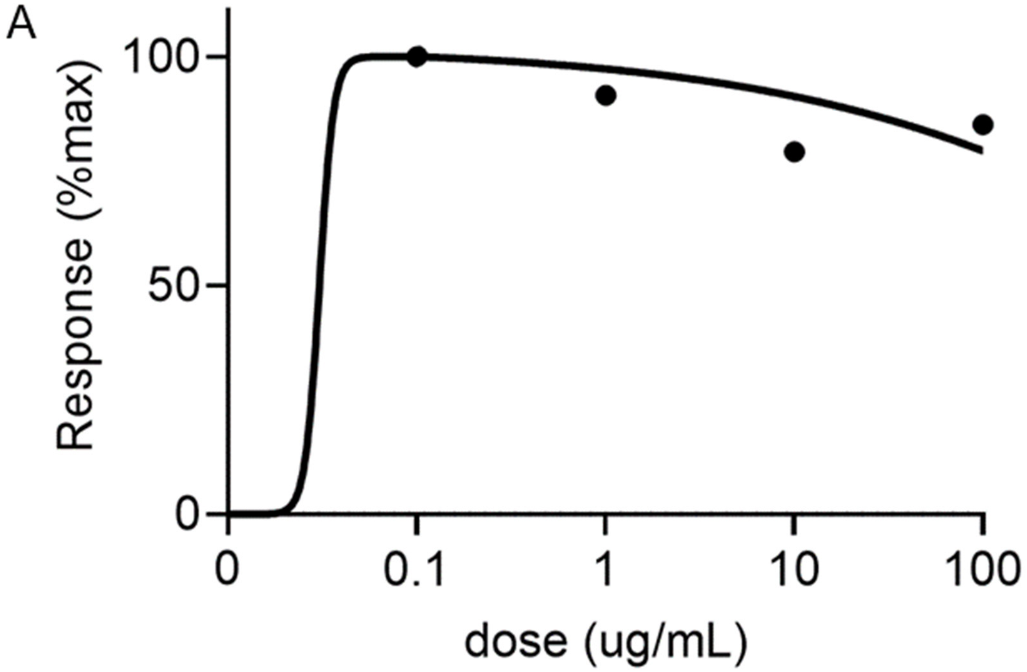

Figure S1. Dose-response curve of HeLa cell viability according to the concentration of ethanol extract of Rhynchosia Volubilis (EERV). Viability of HeLa cells exposed to the control (0.5% (v/v) DMSO(Biosesang)) and EERV (0.1, 1, 10, and 100 μg/mL) for 24 h, as measured using viability assays. The data in Figure 1B was used; the cell viability data of each point was replaced with the response value(%max) and is shown by normalizing the maximum value to 100% and the control value to 0% (EC50=0.05) (n = 6). The absorbance values of solubilized formazan product were measured using the Glomax Multi+Microplate Multi Reader (9301-010, Promega, USA). The graph was generated with GraphPad Prism 7.0 (San Diego, CA).
